# Online Information Behavior Regarding COVID-19 Vaccination and Its Association With Vaccination Behavior Based on Cluster Analysis of User Groups: Cross-Sectional Study

**DOI:** 10.2196/82221

**Published:** 2026-05-29

**Authors:** Lea Liebner, Birgit Babitsch, Lisa Schmidt

**Affiliations:** 1 Department of New Public Health School of Human Sciences Osnabrück University Osnabrück Germany

**Keywords:** cluster analysis, COVID-19 vaccination, information-seeking behavior, internet use, user groups

## Abstract

**Background:**

The COVID-19 pandemic highlighted the importance of effective health communication and reliable information for crisis management, particularly following the introduction of vaccinations. Varied attitudes toward COVID-19 vaccination and an overwhelming amount of online information complicated communication and pandemic management. Previous studies have often focused on general vaccination behavior and its correlation with vaccination attitudes, establishing a link between information-seeking and vaccination decisions. However, there is insufficient analysis distinguishing specific user groups based on their actual online information behavior regarding COVID-19 vaccination and examining its correlation with vaccination behavior.

**Objective:**

This study aims to fill this research gap by identifying user groups based on their information behavior and investigating its influence on vaccination uptake.

**Methods:**

As part of the “Internetnutzung zur COVID-19-Impfung” (INCOVI) study, 1000 individuals in Germany were surveyed online (November 26 to December 8, 2021) regarding their internet usage related to COVID-19 vaccination. A hierarchical cluster analysis was conducted to identify user groups. Logistic regression analyses were then used to explore correlations among the user groups and their demographic characteristics, readiness to vaccinate, knowledge of vaccination, and health literacy. Additionally, a logistic regression analysis was performed to identify the influence of user groups and other factors on vaccination behavior.

**Results:**

A total of 3 user groups were identified: frequent and critical information evaluators (454/778, 58.4%), who primarily relied on official information sources, exhibited a higher level of health literacy, and were older than the other groups; infrequent and passive recipients (222/778, 28.5%), who rarely sought information actively and were younger than the other groups; and frequent and multichannel, interaction-focused users (102/778, 13.1%), who actively searched across multiple channels and engaged in information exchange. Notably, the user groups did not significantly differ in knowledge or willingness to vaccinate. User group affiliation, knowledge, and health literacy did not significantly influence vaccination behavior. The strongest predictor of vaccination was preexisting willingness to vaccinate. Additionally, women were more likely to be vaccinated than men, and individuals with medium or higher education levels were 6-11 times more likely to be vaccinated compared to those with only a basic level of education.

**Conclusions:**

Segmenting the population into different user groups allows for more targeted communication tailored to the specific needs and beliefs of each group. Because these groups stem from observable usage patterns, they constitute a transferable framework for other health topics. For frequent and critical information evaluators, providing well-founded and detailed information on public channels is important. Infrequent and passive recipients benefit from straightforward formats, such as short explanatory videos, while frequent and multichannel, interaction-focused users are better reached through interactive offerings on social media. By specifically targeting these groups, informed decision-making about vaccinations can be supported.

## Introduction

In the context of the COVID-19 pandemic, the importance of effective health communication and informed information behavior became evident worldwide, and in particular in Germany [1]. The rapid introduction of vaccines against SARS-CoV-2 and the overwhelming amount of information necessitated comprehensive and targeted communication to build trust and encourage vaccination. In Germany, vaccination against different diseases is a regular part of health care and is financed by health insurance [2]. However, vaccination rates are below expectations. The SARS-CoV-2 vaccination was the subject of critical discussion, which intensified when it became an obligatory part of the measures to combat the COVID-19 pandemic [3].

The effects of this political shift were also reflected in the vaccination rates. After the introduction of the first COVID-19 vaccines in Germany, the vaccination readiness score (on a scale from 1 to 7) significantly increased from a mean value of 4.14 in December 2020 to 6.45 in December 2021, and then remained consistently high throughout the pandemic [4]. Although willingness to be vaccinated was high, the actual vaccination rate was lower than expected. As of November and December 2021, the basic vaccination rate in Germany was only 69.8%, while the Robert Koch Institute (Germany’s public health institute) aimed for a target vaccination rate of 85% for those aged 12-59 years and 90% for individuals aged 60 years and older by autumn 2021 [5,6]. Consequently, factors other than willingness to be vaccinated influence actual vaccination behavior.

Different models have been developed to explain vaccination behavior. One such is the 7C model by Geiger et al [7], which addresses the mechanism of the preliminary stage of vaccination behavior. It captures the vaccination decisions for or against the COVID-19 vaccination. The 7 “Cs” of the model are: confidence, complacency, constraints, calculation, collective responsibility, compliance, and conspiracy. Confidence refers to an individual’s trust in the efficacy and safety of vaccinations, as well as in the health care system and the responsible organizations. People with high confidence levels are generally more willing to be vaccinated. Complacency is the subjective assessment of one’s own risk of disease. People who perceive their risk of contracting a disease as low often show a lower willingness to be vaccinated. Structural, psychological, or everyday barriers that can make vaccination difficult are summarized in the determinant constraints. If vaccination is not considered particularly important in overcoming barriers, willingness to be vaccinated is low. The determinant calculation refers to the subjective weighing of the risks and benefits or advantages and disadvantages of vaccination for individuals. Collective responsibility describes the willingness to be vaccinated not only to protect oneself, but also to protect others. People with a high sense of responsibility toward the community recognize the indirect protection that their own vaccination offers others and are more willing to be vaccinated. Geiger et al [7] describe the willingness to comply with social recommendations, such as vaccination and monitoring, as compliance [7]. The final determinant of the 7C model, conspiracy, considers the tendency to believe in conspiracy theories and fake news. The 7C model includes psychological, social, and structural factors, making it possible to investigate and understand the multifaceted and complex nature of vaccination behavior. In this study, readiness to vaccinate, as measured by the 7C scale, is considered one of several determinants of vaccination behavior. Another contributing factor could be health information behavior, that is, the way in which people obtain information.

The components of health information behavior can be described by Baumann’s [8] model of information behavior. Baumann [8] assumes that information strategies always exist as multidimensional constructs consisting of different attitudes and behaviors. Individual factors influencing information behavior provide only moderate validity and should not be considered in isolation from one another, as they ultimately interact with each other. At the center of the model is the information strategy, which outlines the specific information behavior of individuals. This behavior is shaped by three dimensions: (1) the aim of the information search—why the individual seeks information, (2) the type of information sought—what topics the person is interested in, and (3) the sources of information—where the person searches. The aims guide how information is handled. Baumann [8] distinguishes between three aims: surveillance, empowerment, and autonomy. The purpose of surveillance is to gain a comprehensive overview of health issues, be informed about risks and diseases, and better understand the health care system. The aim of empowerment includes that patients specifically seek information so that they can actively participate in their medical care; strengthening their own competence should enable them to better classify and evaluate advice and decisions made by health experts. The aim of autonomy goes one step further: here, the patient strives to take on the role of the therapist themselves, that is, to make health-related decisions competently and independently of medical experts, and also to advise others on health issues.

Baumann [8] summarized the topics related to health and illness into four areas: healthy lifestyle/well-being, diseases and treatment options, the health care system, and health care providers/institutions. The sources of information are grouped into 5 categories: general mass media, magazines, academic media, health experts, and the private social environment. Factors influencing these 3 dimensions are the intensity and variety of health-related information searches, the attention given to health topics, and the evaluation of the relevance of the information received for action [8]. In addition, sociodemographic, socioeconomic, and health-related characteristics of individuals impact their information strategy. Baumann’s [8] components of health information behavior are used to form user groups in this study.

When it comes to vaccination behavior, all 3 dimensions of information behavior play an important role: the frequency of information searches, the information channels used, and the reasons for searching for information. Analyses of Google search volume relating to COVID-19 vaccination intentions found that search queries were higher in regions with a large number of people who were still undecided compared to regions with a higher proportion of vaccination enthusiasts [9]. In addition, people who felt poorly informed about COVID-19 vaccines were more likely to refuse vaccination or be undecided [10]. Increased knowledge was associated with targeted independent research and a preference for information from government institutions and health care professionals. At the same time, not using social media was associated with a lower rate of vaccine refusal [10].

So far, most research findings have examined influencing variables separately, with the complex pattern of information seeking, information retrieval, and their impact on behavior being poorly understood in the context of COVID-19 vaccination. User group analyses can be used to make the complex phenomenon of information retrieval comprehensible, segmentable, and manageable. Two studies, Piltch-Loeb et al [11] and Tan et al [12], analyzed user groups concerning health information behavior related to COVID-19 vaccination. Both studies focused on the third dimension of Baumann’s [8] model, the source of information and its relationship with vaccine intentions or status. Using a sample of 3014 Americans, Piltch-Loeb et al [11] defined 6 types based on their use of traditional media, social networks, and vaccination attitudes. For example, users who primarily relied on Facebook and Instagram, as well as nonseekers, were less likely to be vaccinated and held more negative attitudes toward vaccination. Tan et al [12] identified 4 types based on trust in information sources of Singaporeans aged 56-75 years (N=6094). High trust in official sources, such as health authorities and scientific institutions, correlated with a higher likelihood of vaccination, while distrust in all sources was associated with a lower likelihood of vaccination. These findings underscore the importance of trust in the credibility of information sources, indicating that health information behavior can influence vaccination behavior [13,14].

A nuanced understanding of specific user groups—defined by their online information-seeking behavior (frequency of information searches, information channels used, and the reasons for searching for information)—remains lacking. Identifying these groups and their characteristics is crucial for tailoring effective public health communication strategies. Therefore, this study seeks to identify distinct user groups based on their online information behavior regarding COVID-19 vaccination and to investigate the key factors known to influence vaccination uptake in Germany, such as demographics, health literacy, and vaccination readiness (7Cs). The following research questions are explored:

1. Which user groups can be identified concerning information behavior regarding COVID-19 vaccination on the internet within the German population?
2. What relationships exist among sociodemographic characteristics, vaccination readiness, knowledge about COVID-19 vaccination, health literacy, and membership in a user group?
3. To what extent does membership in a user group correlate with vaccination status?

In addition to vaccination readiness and information behavior, other factors can influence vaccination behavior, such as health literacy. Good health literacy enables individuals to find and understand information and make decisions accordingly [15,16]. Furthermore, sociodemographic characteristics such as age, gender, education, income, and immigration history have been highlighted as significant influencing factors [11,15-17]. In particular, a higher level of education and greater health literacy have been associated with more positive attitudes toward vaccination [11,12]. Additionally, specific knowledge about COVID-19 vaccination can also affect vaccination behavior [18]. According to Tan et al [12], factors such as place of residence and apartment size also correlate with vaccination behavior. Self-efficacy expectations can also play a role, as individuals with high self-efficacy are more likely to overcome barriers and choose preventive measures such as vaccination [19]. Conversely, those who feel disproportionately high external pressure to be vaccinated exhibit lower willingness to vaccinate against COVID-19 [20]. This study also aims to examine which of the aforementioned factors influenced vaccination behavior, in particular membership of a user group. Therefore, the third research question is: “To what extent does membership in a user group correlate with vaccination status?” Accordingly, the following hypotheses are proposed:

Hypothesis 1: There is a correlation between identified user groups and sociodemographic characteristics, willingness to vaccinate, knowledge about COVID-19 vaccination, and health literacy.

Hypothesis 2: Belonging to an identified user group predicts vaccination status.

## Methods

### Study Design

Data were collected using an online questionnaire from November 26 to December 8, 2021, by an external provider of online surveys (Bilendi GmbH). This period coincided with increased media coverage of the first booster vaccinations for COVID-19. The sample was a quota sample, representative of age, gender, and place of residence distribution within a single federal state according to Eurostat 2018. Bilendi invited registered users to participate, offering remuneration for completion. The invitation included information on survey duration, compensation, and a link to the questionnaire. Eligible participants were German-speaking individuals aged 18-75 years.

The questionnaire was divided into three sections: (1) internet use for COVID-19 vaccination information, knowledge about the vaccination, and health literacy; (2) attitudes toward vaccination, vaccination behavior, and determinants of vaccine willingness; and (3) sociodemographic characteristics.

### Ethical Considerations

This research project was approved by the Ethics Committee of Osnabrück University (reference Ethik-46/2021 and Ethik-68/2021). Bilendi ensured data protection and anonymity in accordance with the German Data Protection Act. The participants were informed about the method of data collection, anonymized data transfer, and compensation during panel registration, and were assured that their responses would remain anonymous and not be shared with third parties. Participation was voluntary and could be terminated at any time without penalty. The participants accessed the survey at Respondi Mingle [21] after accepting the terms and conditions and data protection guidelines.

### Online Usage Behavior for COVID-19 Vaccination

The frequency of using the internet to search for information regarding COVID-19 vaccination was recorded using the following items: daily, several times a week, once a week, less than once a week, not at all, and use of other sources.

Channels used to search for information: the participants were asked about the most common online information channels. The responses were initially coded as follows: perceived fleetingly, consciously searched for information, no contact, and do not use. Since the options “no contact” and “I don’t use” were similar, these were combined into “consciously searched for information,” “fleetingly perceived,” and “no contact/I don’t use.”

Formats: the participants were asked which formats related to COVID-19 vaccination they encountered online (text articles, audio articles, videos, posts on social networks, posts via messenger services, discussion forums), using a scale of frequently, sometimes, rarely, and never.

Reasons for information-seeking: the reasons for seeking information about COVID-19 vaccination online included obtaining in-depth information about the vaccination, researching information, or exchanging views with like-minded individuals about the benefits and risks of the vaccination. The participants confirmed these statements with “yes” or rejected them with “no.”

### Sociodemographic Characteristics

Age was categorized into 4 groups: 18-29 years, 30-44 years, 45-64 years, and 65-75 years. Education was recorded based on school education and professional qualifications according to the demographic standard of the Federal Statistical Office of Germany [22]. Education levels were then clustered into basic, medium, and higher educational attainment using the Comparative Analysis of Social Mobility in Industrial Nations classification [23]. Immigration history was assessed according to the recommended standards in Germany [24,25]. A distinction was made between two categories: (1) immigrants and their (direct) descendants (ie, participants not born in Germany or participants born in Germany with both parents born outside of Germany) and (2) individuals without an immigration history (ie, participants and both parents born in Germany) [26]. Place of residence was categorized into rural areas (up to 5000 inhabitants), small towns (5001 to under 20,000 inhabitants), medium-sized cities (20,000 to 100,000 inhabitants), and large cities (more than 100,000 inhabitants) [27].

### Health Literacy

A total of 6 items on general health literacy were developed based on Schaeffer et al’s [28] study. Participants answered the following questions: When you think back to the time before the coronavirus pandemic, how easy was it for you to (1) obtain health-related information, (2) comprehend health-related information, (3) evaluate the content of health-related information, (4) assess the credibility of health-related information, (5) critically question health-related information, and (6) use health-related information as a basis for decision-making (translation from German). The answer options from the Likert scale were coded as follows: 1=very difficult, 2=difficult, 3=neither, 4=easy, 5=very easy. By summing the results, a new variable, “health literacy score,” was created (range from 6 [minimum] to 30 [maximum]). The interitem correlation for this survey is α=.86.

### Knowledge

For the knowledge question on COVID-19 vaccination, 5 statements were developed based on information or vaccination myths published on the Bundesministerium für Gesundheit website [29].

Please evaluate the following statements: (1) An mRNA vaccine can alter human DNA. (2) Vaccine reactions such as pain at the injection site, fatigue, headache, and muscle pain are signs that the immune system is responding to the vaccine as intended. (3) Depending on the vaccine, vaccinated individuals are 65%-95% less likely to contract COVID-19 than those who are not vaccinated. (4) After vaccination, I no longer have to follow the rules of conduct (keeping distance, wearing a mask). (5) Full immunity is achieved immediately after vaccination.

A total score (knowledge score from 0 to a maximum of 5 points) was calculated with values for each item as either “not known” (0) or “known” (1).

### External Pressure on Vaccination Behavior

The participants were asked whether they felt external pressure to be vaccinated, for example, from their employer or the general population, or whether they felt free to decide for or against vaccination (1 item, possible answers: yes/no).

### Self-Efficacy Expectations

Self-efficacy was measured using the current version of the validated General Self-Efficacy Scale by Jerusalem and Schwarzer [30]. The self-assessment procedure comprises 10 items and is used to record general optimistic self-beliefs. The interitem correlation for this survey is α=.92.

### Vaccination Readiness and Vaccination Status

The validated 7C Vaccination Readiness Scale by Geiger et al [7] was used to assess readiness to vaccinate, evaluated as recommended by the authors. The scale contains 7 components of readiness to vaccinate: confidence, complacency, constraints, calculation, collective responsibility, compliance, and conspiracy, each with 3 associated items (range: 1=strongly disagree to 7=strongly agree). The instrument is publicly available [31]. Readiness to vaccinate is represented as the average value across all items of the 7C scale. The internal consistency of the scale was examined using McDonald’s omega (ω). The value of ω=0.96 indicates very good internal consistency.

For vaccination status, it was first determined whether the individuals had received 1 or 2 vaccine doses (from BioNTech/Pfizer, Moderna, or AstraZeneca) or 1 vaccine dose from Johnson & Johnson or none to date, which corresponded to the vaccination recommendation in Germany. This information was then summarized into 2 categories—vaccinated or unvaccinated—to represent the participants’ vaccination status.

### Statistical Analyses

Analyses on online information behavior were only conducted with a group of 889 participants who searched online for information about the COVID-19 vaccination. Absolute and relative frequencies were calculated for the formats and channels used, the reasons for searching for information about COVID-19, and the frequency of searching for information on the internet over the last 4 weeks from completing the questionnaire. A cluster analysis was conducted to determine user groups regarding information behavior about COVID-19 vaccination online. The channels, formats, frequency of information behavior, and reasons for searching for information about the vaccination were used as potential variables for determining user groups through a cluster analysis.

Before the cluster analysis, a Spearman correlation analysis was conducted between the ordinal-scaled variables. Significant, strong correlations were identified between the variables channels and formats (podcast channel and audio contributions: Spearman ρ=0.58, *P*<.001; WhatsApp and posts via messenger services: Spearman ρ=0.67, *P*<.001; Facebook and posts in social networks: Spearman ρ=0.63; *P*<.001). Consequently, the format variables were excluded, which can also be justified because they lacked variation. For the cluster analysis, a hierarchical cluster analysis using complete linkage and Gower distance was applied in RStudio (Posit PBS). After removing cases with missing values and outliers (identified through single-linkage analysis) from the group of 889 participants searching for information online, 778 cases were included in the cluster analysis. The optimal number of clusters was determined using the elbow method and the silhouette method, both of which indicated that 2- and 3-cluster solutions were appropriate. Based on content-related considerations, the 3-cluster solution was selected. A subsequent discriminant analysis showed that 86.4% (672/778) of the originally grouped cases were correctly classified, indicating high quality and accuracy of the cluster analysis and supporting the decision to adopt the 3-cluster model.

Bivariate analyses (chi-square test and ANOVA) were conducted to identify differences between user groups. To analyze the correlations between the user groups and personal characteristics, a binary logistic regression was performed with 3 models for each user group. Model 1 included sociodemographic characteristics (gender, age, education, immigration history, and size of place of residence). Model 2 added willingness to vaccinate, and model 3 incorporated knowledge about vaccination and health literacy. 

Additionally, a binary logistic regression analysis was conducted to predict vaccination status. In the first model, the correlation with user groups was tested. The second model included sociodemographic variables, while the third model added potential influencing factors such as vaccination readiness score, knowledge, health literacy, external pressure, and self-efficacy expectation. These analyses were performed using IBM SPSS Statistics, version 29.

## Results

### Description of the Sample

In total, 1000 people living in Germany completed the questionnaire. Among the participants, 50% (n=500) identified themselves as male and 50% (n=500) as female. The average age was 46.4 (SD 15.5) years, with ages ranging from 18 to 75 years. Further sociodemographic characteristics of the study population revealed that 8.6% (n=86) had a history of immigration. In terms of educational attainment, 12.9% (n=129) had a basic level, 57.8% (n=578) had a medium level, and 27.1% (n=271) had a higher level. More than half of the respondents lived in a medium-sized town or a large city. Overall, 86% (n=860) of the participants had been vaccinated at least once, while 13.3% (n=133) remained unvaccinated at the time of the survey. Attitudes toward vaccination were as follows: 13.7% (n=137) were (somewhat) opposed, 6.9% (n=69) were undecided, and 78.7% (n=787) were (somewhat) in favor. An overview is provided in Table 1.

**Table 1:** Description of the sample (N=1000).

Characteristic		Value
**Age (years), n (%)**		
	18-29	188 (18.8)
	30-44	271 (27.1)
	45-64	403 (40.3)
	65-75	138 (13.8)
**Gender, n (%)**		
	Male	500 (50)
	Female	500 (50)
**Immigration history, n (%)**		
	Individuals without an immigration history	914 (91.4)
	Immigrants and their (direct) descendants	86 (8.6)
**Education attainment, n (%)**		
	Basic	129 (12.9)
	Medium	578 (57.8)
	Higher	271 (27.1)
	Prefer not to answer	22 (2.2)
**Residence, n (%)**		
	Rural area	202 (20.2)
	Small town	191 (19.1)
	Medium-sized city	247 (24.7)
	Big city	353 (35.3)
	Prefer not to answer	7 (0.7)
**Vaccination status, n (%)**		
	Vaccinated at least once	860 (86)
	Not vaccinated	133 (13.3)
	Prefer not to answer	7 (0.7)

### Online Information Behavior

In the past 4 weeks, most respondents (N=1000) reported searching the internet for information on COVID-19 vaccination daily (n=266, 26.6%) or several times a week (n=356, 35.6%). Conversely, 11% (n=110) did not search online at all for the information or used other sources.

**Figure 1:**
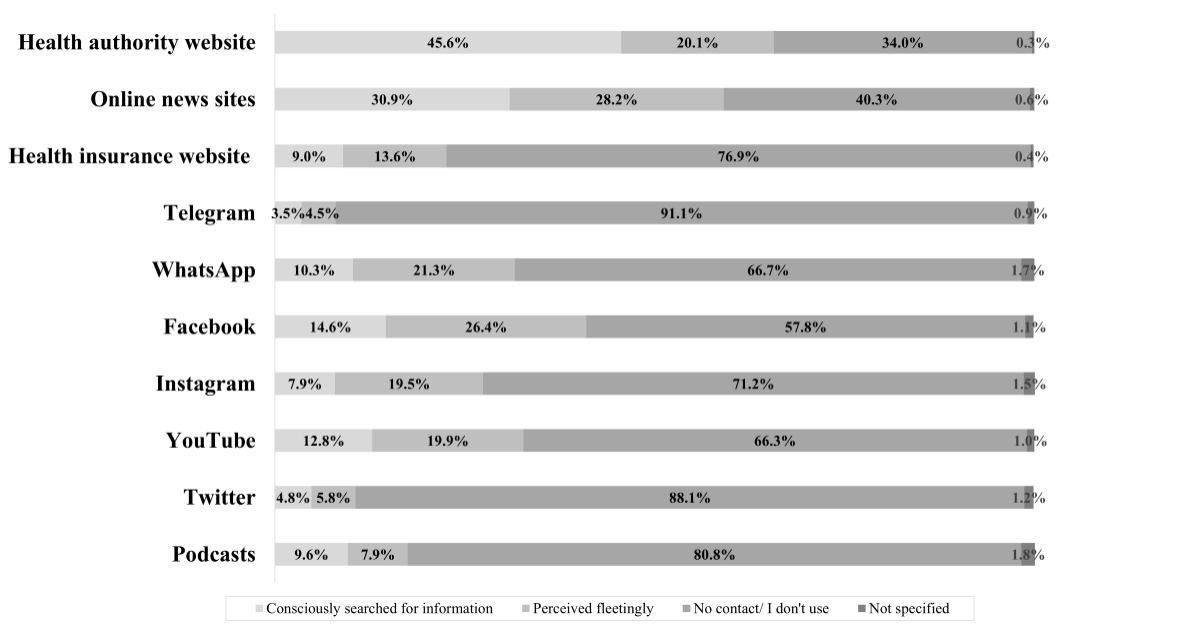
Channels used to search for information (n=889).

Further analyses of information behavior were conducted only with the subsample of 889 online users. Websites of health authorities (n=405, 45.6% consciously searched) and online news sites (n=275, 30.9% consciously searched) were used more frequently than health insurance company websites (80/889, 9%). Social media and messenger services were used less intentionally for information gathering. Facebook, WhatsApp, and Instagram were perceived as less reliable sources, while Telegram and Twitter were used by only a few online users (Figure 1).

Text content was the most frequently selected method for learning about COVID-19 vaccination (405/889, 45.6% often and 312/889, 35.1% sometimes). Posts on social media and audio and video content were used less frequently, while discussion forums and messages via messenger services were rarely selected (Multimedia Appendix 1).

Of the 889 online users, 85.9% (n=764) stated that their reason for searching for information on COVID-19 vaccination was to stay updated on new developments. Additionally, 59.4% (n=528) sought to learn more about the vaccination, and 69.4% (n=617) wanted to verify information and research claims. A less substantial reason for using the internet was the intention to persuade others of their views on vaccination through social media posts (131/889, 15.1%). Furthermore, 43.6% (388/889) of respondents reported regularly discussing the advantages and disadvantages of vaccination with others, and half sought information outside of public institutions.

### Identified User Groups on Information Behavior Regarding COVID-19 Vaccination on the Internet

#### Overview

Due to missing values, only 778 participants could be included in the cluster analysis. We identified 3 distinct user groups: “frequent and critical information evaluators,” “*infrequent* and passive recipients,” and “frequent and multichannel, interaction-focused users.” The distribution of user behavior, personal characteristics, and attitudes among these groups is detailed below (Table 2). The bivariate analyses revealed significant differences between the user groups in terms of age, self-efficacy, knowledge, and health literacy.

**Table 2:** Distribution of sociodemographic characteristics, vaccination behavior, vaccination attitudes, health literacy, and knowledge among the user groups and differences between groups (n=778).

Variable			User group 1 (n=454)		User group 2 (n=222)		User group 3 (n=102)		Differences between groups			*P* value	
**Gender, n (%)**										4.51 (2)^a^			.11
	Male	220 (48.5)		125 (56.3)		57 (55.9)							
	Female	234 (51.5)		97 (43.7)		45 (44.1)							
**Age groups (years), n (%)**										12.71 (6)^a^			.048
	18-29	85 (18.7)		49 (22.1)		21 (20.6)							
	30-44	110 (24.2)		62 (27.9)		35 (34.3)							
	45-64	182 (40.1)		91 (41)		35 (34.3)							
	65-75	77 (17)		20 (9)		11 (10.8)							
**Educational attainment, n (%)**										3.53 (4)^a^			.47
	Basic	48 (10.7)		25 (11.5)		13 (13.1)							
	Medium	250 (55.7)		133 (61)		59 (59.6)							
	Higher	151 (33.6)		60 (27.5)		27 (27.3)							
**Immigration history, n (%)**										3.63 (2)^a^			.16
	Immigrants and their (direct) descendants	47 (10.4)		17 (7.7)		5 (4.9)							
	Individuals without an immigration history	407 (89.6)		205 (92.3)		97 (95.1)							
**Residence, n (%)**										5.49 (6)^a^			.48
	Rural area	83 (18.3)		49 (22.1)		14 (13.7)							
	Small town	88 (19.4)		37 (16.7)		22 (21.6)							
	Medium-sized city	122 (24.7)		61 (27.5)		24 (23.5)							
	Big city	170 (37.5)		75 (33.8)		42 (41.2)							
**Vaccination status, n (%)**										3.31 (2)^a^			.19
	Vaccinated at least once	410 (90.3)		195 (87.8)		86 (84.3)							
	Not vaccinated	44 (9.7)		27 (12.2)		16 (15.7)							
**Attitude toward vaccination, n (%)**										6.19 (4)^a^			.19
	(Rather) negative	42 (9.3)		28 (12.6)		15 (14.7)							
	Undecisive	26 (5.8)		17 (7.7)		3 (2.9)							
	(Rather) favorable	384 (85)		177 (79.7)		84 (82.4)							
**Decision to vaccinate without external pressure, n (%)**										1.77 (2)^a^			.41
	Yes	382 (85.1)		181 (81.5)		83 (83.6)							
	No	67 (14.9)		41 (18.5)		19 (18.6)							
Self-efficacy, mean (SD)			30.44 (5.56)		29.85 (5.06)		32.12 (5.87)		5.64 (2, 721)^b^			.004	
Knowledge on COVID-19 vaccination, mean (SD)			4.34 (0.99)		4.09 (1.03)		4.05 (1.10)		6.35 (2, 767)^b^			.002	
Health literacy, mean (SD)			21.57 (4.18)		20.29 (4.13)		22.66 (4.64)		12.13 (2, 757)^b^			<.001	

^a^Chi-square (*df*).

^b^*F* test (*df*).

#### Frequent and Critical Evaluators (User Group 1)

A total of 58.4% (n=454) of the participants were categorized into this group. They searched the internet for information about COVID-19 vaccination several times a week (n=199, 43.8%) or daily (n=158, 34.8%) during the 4 weeks prior to completing the questionnaire. They also deliberately used public channels, such as health authority websites (n=240, 52.9%). Social media platforms—such as Facebook (n=46, 10.1%), Instagram (n=24, 5.3%), and Twitter (n=26, 3.5%), as well as messenger services such as WhatsApp (n=31, 6.8%) and Telegram (n=6, 1.3%)—were rarely used for information. YouTube and podcasts were also infrequently used (n=39, 8.6% and n=40, 8.8%, respectively). Key reasons for internet use regarding COVID-19 vaccination for this group included obtaining detailed information (n=360, 79.3%), verifying information and research claims (n=360, 79.3%), and staying informed on current developments (n=445, 98%).

In the logistic regression analysis, participants older than 45 years with higher education levels were more likely to belong to this user group. Overall, 85% (n=384) of this group were (somewhat) in favor of vaccination, and 90.3% (n=410) had been vaccinated at least once. On average, user group 1 scored higher in knowledge than the overall participant group (mean 4.34, SD 0.99 compared with mean 4.23, SD 1.02) and rated their health literacy higher (mean 21.57, SD 4.18 compared to mean 21.37, SD 4.29). The probability of being assigned to user group 1 was 1.6 times higher for women than for men (*P*=.007). No further significant correlations could be identified with other sociodemographic characteristics, willingness to be vaccinated, knowledge, or health literacy.

#### Infrequent and Passive Recipients (User Group 2)

This group comprised 28.5% (n=222) of the participants. They searched for information on the internet less frequently than the other 2 user groups, with 20.7% (n=46) searching once a week and 36% (n=80) doing so less than once a week. Members of user group 2 intentionally searched for information less frequently, regardless of the channel. Regarding their reasons for using the internet in relation to COVID-19 vaccination, the only reason that garnered agreement from more than half of the respondents was the desire to stay informed about the latest developments regarding the vaccination (n=136, 61.3%). This user group was characterized by occasional information searches and a passive attitude toward seeking information online about COVID-19 vaccination.

Individuals aged 18-29 years and 30-44 years, particularly those with a medium level of education, were more likely to belong to this user group. Additionally, this group contained a higher proportion of unvaccinated individuals and those undecided about vaccination. Overall, 79.7% (n=177) of the participants in user group 2 were (somewhat) in favor of vaccination, and 87.8% (n=195) had been vaccinated at least once.

Members of this user group rated their health literacy lower than those in the other 2 groups (mean 20.29, SD 4.13), which was also reflected in their knowledge of COVID-19 vaccination (mean 4.09, SD 1.03). In the logistic regression analysis, a significant correlation was found only for user group 2 concerning health literacy and age groups. Higher health literacy was associated with a lower probability of being assigned to user group 2 (odds ratio [OR] 0.93, 95% CI 0.89-0.96; *P*<.001). People aged 65 years and older were less likely to be in user group 2 than 18- to 29-year-olds (OR 0.41, 95% CI 0.21-0.78; *P*=.01).

#### Frequent and Multichannel, Interaction-Focused Users (User Group 3)

This group accounted for 13.1% (n=102) of the participants. During the 4 weeks prior to completing the questionnaire, 48% (n=49) of individuals searched for information about COVID-19 vaccination on the internet daily, while 43.1% (n=44) did so several times a week. Members of this user group actively sought information from online news sites (n=66, 64.7%) and public channels, such as health authority websites (n=62, 60.8%), as well as social media platforms such as Facebook (n=43, 42.2%), YouTube (n=46, 45.1%), and the WhatsApp messaging service (n=38, 37.3%). They rarely used channels such as Telegram, Twitter, and Instagram. The most common reasons for using the internet regarding COVID-19 vaccination included following current developments (n=102, 100%), obtaining in-depth information about the vaccination (n=98, 96.1%), researching statements and verifying information (n=99, 97.1%), regularly exchanging information with like-minded individuals about the benefits and risks of vaccination (n=91, 89.2%), and seeking information outside of public institutions (n=81, 79.4%). The information behavior of this user group overlaps with that of the frequent and critical information evaluators; however, their choice of diverse channels and intention to exchange information with others was particularly notable.

Individuals aged 30-44 years were increasingly represented in this group. Overall, 82.4% (n=84) of participants in this user group were (rather) in favor of vaccination, and 84.3% (n=86) had been vaccinated at least once. Although members of this group rated their health literacy higher than those in the other two groups (mean 22.66, SD 4.64), they achieved the lowest average score on the knowledge questions (mean 4.05, SD 1.10).

In the logistic regression analysis, a modest association was observed between health literacy and assignment to this user group. As health literacy increased, the probability of being assigned to user group 3 slightly increased (OR 1.08, 95% CI 1.02-1.13; *P*=.01). No statistically significant correlation was found between other characteristics and the vaccination variables.

The regression models can be found in the Multimedia Appendix 2.

### Predictors of Vaccination Status

A logistic regression was performed to identify the factors influencing vaccination status (Table 3). Model 1 indicated that the user group is not related to vaccination status. When personal characteristics were added in model 2, education and place of residence emerged as significant influencing factors. Model 3 demonstrated that vaccination readiness—defined as personal attitudes toward COVID-19 vaccination—was the most important predictor of vaccination status. For each additional point on the Vaccination Readiness Scale, there is a 9.72 (95% CI 5.68-16.66; *P*<.001) times higher chance of being vaccinated. Another statistically significant influencing factor remains education; compared to people with a basic level of education, those with a medium level of education have a 6.78 (95% CI 1.76-26.15; *P*=.005) times higher chance, and people with a higher level of education have an 11.90 (95% CI 2.39-59.18; *P*=.002) times higher chance of being vaccinated. In addition, men have a 65% (95% CI 14%-86%; *P*=.02) lower chance of being vaccinated than women. Knowledge about vaccination, health literacy, external pressure, and self-efficacy did not play a significant role in the overall model.

**Table 3:** Logistic regression to predict vaccination status (reference value: vaccinated at least once; N=1000).

Variable		Model 1		Model 2		Model 3			
		*P* value	OR^a^ (95% CI)	*P* value	OR (95% CI)	*P* value	OR (95% CI)		
**User group**									
	User group 1	—^b^	1	—	1	—	1		
	User group 2	.31	0.75 (0.44-1.30)	.61	0.86 (0.49-1.52)	.56	1.34 (0.49-3.67)		
	User group 3	.27	0.67 (0.34-1.35)	.44	0.75 (0.37-1.55)	.77	1.21 (0.34-4.23)		
**Age group (years)**									
	18-29	—	—	—	1	—	1		
	30-44	—	—	.93	1.03 (0.52-2.03)	.54	1.44 (0.45-4.65)		
	45-64	—	—	.14	1.67 (0.84-3.33)	.07	2.99 (0.92-9.73)		
	65-75	—	—	.06	2.65 (0.95-7.39)	.62	1.68 (0.21-13.36)		
**Gender**									
	Female	—	—	—	1	—	1		
	Male	—	—	.32	0.77 (0.46-1.29)	.02	0.35 (0.14-0.86)		
**Education attainment**									
	Basic	—	—	—	1	—	1		
	Medium	—	—	.06	1.97 (0.97-4.01)	.01	6.78 (1.76-26.15)		
	Higher	—	—	.002	4.01 (1.69-9.53)	.002	11.9 (2.39-59.18)		
**Immigration history**									
	Immigrants and their (direct) descendants	—	—	—	1	—	1		
	Individuals without immigration history	—	—	.61	0.8 (0.34-1.88)	.21	2.46 (0.60-10.07)		
**Residence**									
	Rural area	—	—	—	1	—	1		
	Small town	—	—	.54	1.25 (0.61-2.58)	.10	0.32 (0.08-1.24)		
	Medium-sized city	—	—	.33	1.4 (0.71-2.78)	.81	0.86 (0.24-3.06)		
	Big city	—	—	.01	2.47 (1.23-4.95)	.80	1.17 (0.34-3.98)		
Vaccination readiness (scale)		—	—	—	—	<.001	9.72 (5.68-16.66)		
Knowledge score		—	—	—	—	.92	0.98 (0.60-1.58)		
Health literacy score		—	—	—	—	.16	0.93 (0.83-1.03)		
**Decision to vaccinate without external pressure**									
	Yes	—	—	—	—	—	1		
	No	—	—	—	—	.41	1.43 (0.61-3.38)		
Self-efficacy (score)		—	—	—	—	.79	0.99 (0.92-1.07)		

^a^OR: odds ratio.

^b^Not available.

## Discussion

### Principal Findings and Comparison With Prior Work

In our study, we observed different information behavior regarding the COVID-19 vaccination, indicating not only the use and nonuse of such information but also the role of internet- and social media–based information. According to Baumann’s [8] model of information behavior, the groups were formed based on search frequency, motivation for searching, and channels used. The 3 user groups identified through cluster analyses are clear examples of cumulative strategies. The frequent and critical information evaluators (user group 1) rely on internet-based information of public authorities, whereas for the interaction-focused users (user group 3), social media–based information plays an important role. Furthermore, the infrequent and passive recipients reflect population groups (user group 2) who use information on COVID-19 vaccination less frequently. The observed types were similar to those described by Tan et al [12] and Piltch-Loeb et al [11], suggesting a degree of consistency in the results. Note that different variables were used in these studies to form the clusters and identify influencing factors. Frequent and critical information evaluators exhibited similar information behavior to the user groups “legacy” and “traditional omnivores” in Piltch-Loeb et al’s [11] study and “pro-formal selective” and “broad-trust” in Tan et al’s [12] study. Each of these user groups increasingly relied on local, traditional, and public channels from authorities to search for information, while making less use of social media and messaging services. In terms of vaccination behavior and attitudes, individuals in these groups were generally more willing to be vaccinated and demonstrated higher vaccination rates [11]. People with a strong intention to vaccinate and a positive attitude toward vaccination tend to use official websites more frequently, engage in more thorough information-seeking, and conduct searches more often [15,16].

There are parallels between the infrequent and passive recipients and the "nonseekers" in Piltch-Loeb et al’s [11] study or "broad distrust" in Tan et al’s [12] study. These groups rarely, if ever, actively searched for information and either did not use any channels or lacked trust in them. Consequently, these groups exhibited a low probability of vaccination and an ambivalent attitude toward COVID-19 vaccination.

The frequent and multichannel, interaction-focused users shared similarities with the "omnivore" + "broad social media users" and the "legacy and Facebook/Instagram users" [11]. Individuals in these user groups sought information about COVID-19 vaccination from both traditional public sources, such as government authorities, and increasingly from social media platforms such as Facebook and Instagram, as well as messaging services like WhatsApp [11]. Additionally, these user types tended to exhibit a higher likelihood of being unvaccinated and often held an undecided or negative attitude toward vaccination [11,12]. This trend may be linked to the observation that individuals who rely more on social media platforms for information rather than official websites generally have lower vaccination rates and a more negative view of vaccination [15,16]. The quality of information on these social media sources is often heterogeneous, and misinformation can spread rapidly, leading to uncertainty, skepticism, and rejection [32,33].

Frequent and critical information evaluators predominantly consisted of participants older than 44 years, who increasingly sought information about COVID-19 vaccination from traditional public and official sources [11]. In contrast, respondents aged 18-29 years were more likely to belong to the infrequent and passive recipients group. Individuals in this age bracket tend to engage in minimal or no active searching for information; when they do seek information, they adopt a targeted and specific approach [8,11].

The results of the analysis indicate that people with higher health literacy (user groups 1 and 3) searched for information on the internet more frequently than people with lower health literacy (user group 2). These are particularly groups who state that the reason for their search is to find out about current developments and to verify information. The study by Moffett et al [9] showed that people who are undecided about vaccination are more likely to conduct online research. This could not be confirmed in our study. However, a kind of uncertainty aspect may also have played a role among the frequent users identified here.

Notably, no clear correlation was identified between the user groups and vaccination status. Consequently, the determinants of information seeking identified by Baumann [8]—frequency, channels, and motivations underlying information-seeking activities—do not seem to be the primary determinants in this context. Instead, preliminary evidence suggests that the quality of the information accessed and the trust placed in it may influence vaccination behavior—variables that were not addressed in this investigation. Furthermore, only a few social differences could be observed, namely in gender and education. The vaccination readiness emerged as the strongest predictor of vaccination behavior in the analysis presented, highlighting the significance of individual beliefs and attitudes. These findings are consistent with previous studies that emphasized risk perception and social influence on vaccination behavior, which are components of the 7C Vaccination Readiness Scale used in this study [7,20].

### Limitations

Overall, when interpreting the results, it is important to consider that the study’s sample exhibited a higher vaccination rate of 86% (860/1000) compared to the 70% vaccination rate reported by the Robert Koch Institute for all citizens in Germany at the time of the survey [5]. Furthermore, the method of participant selection—the sample’s distribution—can also explain the discrepancies between the study’s findings and existing theories regarding the factors influencing information-seeking and vaccination behavior. The survey was conducted online by an external provider, which limited participation to individuals with internet access and registration with that provider. This limitation may impact representativeness, as those who are online are more likely to search for health information. Additionally, the health literacy score was based on subjective assessments, potentially leading to bias due to overestimation by the respondents. Moreover, no causal conclusions can be drawn from the cross-sectional design.

### Recommendations

Several recommendations can be made for health communication regarding COVID-19 vaccination that may also apply to future novel infectious diseases. Identifying distinct types of online information use allows for a more nuanced understanding of information behavior. Targeted health communication to different user groups may not influence the vaccination behavior, but could lead to an informed vaccination decision, for example, understanding the risks and benefits. The following recommendations support a targeted approach for providing relevant health information to the identified user groups. For frequent and critical information evaluators, more evidence-based and detailed information could be provided on government and scientific platforms. Infrequent and passive recipients could be more effectively reached through low-threshold, easily understandable formats. In contrast, interactive formats, such as those found in social media or discussion forums, could better engage frequent and multichannel, interaction-focused users. Because this group obtains information from platforms that historically exhibit lower information accuracy, public health agencies should implement targeted interventions. For example, collaboration with trusted microinfluencers and platform‑native content creators could help coproduce short, evidence‑based messages that match the stylistic norms of each channel.

The 3 information-seeking groups identified in our study reflect fundamental behavioral patterns in how individuals obtain and process health information. Because these clusters were derived from usage‑based variables, they have the potential to serve as a generalizable framework for other health‑related issues. However, the transferability of the cluster structure is not automatic. For each new health topic, researchers should collect the same behavioral variables, conduct a confirmatory cluster or latent‑class analysis to test whether the original 3‑class solution fits the new data, and examine how cluster membership relates to the specific health behavior of interest. When the clusters are confirmed, targeted communication strategies that proved effective for COVID‑19 vaccination can be adapted to the new context.

Longitudinal studies are required to assess whether communication strategies that are tailored to each group produce sustained changes in knowledge, attitudes, and subsequent health actions. Finally, the efficacy of the proposed communication strategies should be evaluated through randomized controlled trials, allowing for evidence‑based optimization of future communication campaigns.

## Data Availability

The datasets generated or analyzed during this study are available from the corresponding author upon reasonable request.

## Appendix

Multimedia Appendix 1 Formats used for information (N=889).

Multimedia Appendix 2 Logistic regression analyses on the characteristics of the user groups.

## Abbreviations

OR: odds ratio
