# Supplementary figures and images for: Online Information Behavior Regarding COVID-19 Vaccination and Its Association With Vaccination Behavior Based on Cluster Analysis of User Groups: Cross-Sectional Study
Source: JMIR Infodemiology. 2026 May 29;6:e82221. doi: 10.2196/82221 (PMC13263656; doi:10.2196/82221)

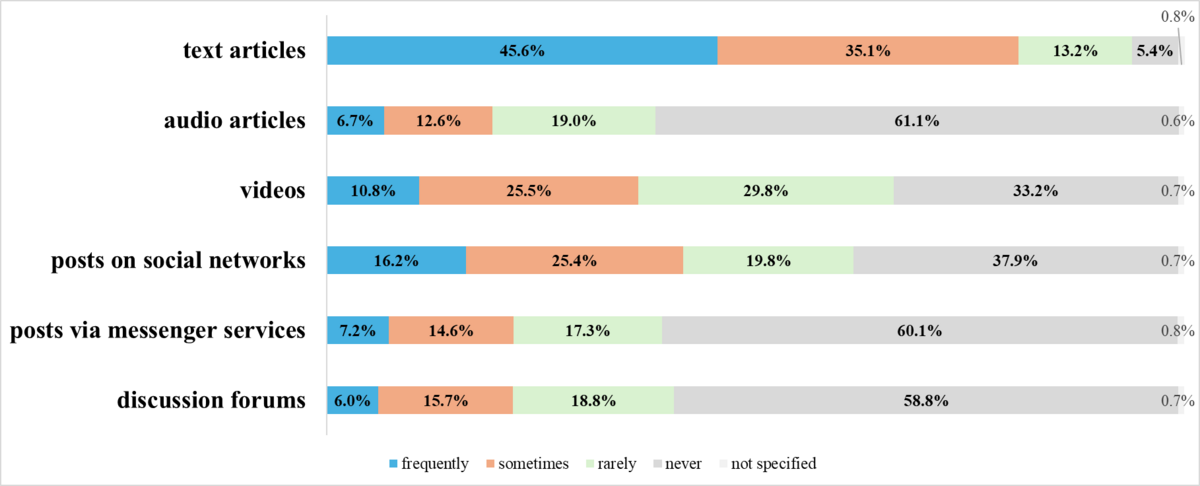

Supplement: Multimedia Appendix 1 [file infodemiology_v6i1e82221_app1.png]
