# Supplementary material for: Online Information Behavior Regarding COVID-19 Vaccination and Its Association With Vaccination Behavior Based on Cluster Analysis of User Groups: Cross-Sectional Study
Source: JMIR Infodemiology. 2026 May 29;6:e82221. doi: 10.2196/82221 (PMC13263656; doi:10.2196/82221)
Supplement: Multimedia Appendix 2 [file infodemiology_v6i1e82221_app2.docx]

**Logistic regression analyses on the characteristics of the user groups**

Logistic regression on the characteristics of the *Frequent and Critical Information Evaluators*

| Variable | | Model 1 | | Model 2 | | Model 3 | |
| --- | --- | --- | --- | --- | --- | --- | --- |
|  | | *P value* | OR (95% CI) | *P value* | OR (95% CI) | *P value* | OR (95% CI) |
| **Age group (years)** | |  |  |  |  |  |  |
|  | 18-29 | — | 1 | — | 1 | — | 1 |
|  | 30-44 | .98 | 1.01 (0.65-1.57) | .95 | 1.01 (0.65-1.58) | .86 | 1.04 (0.66-1.63) |
|  | 45-64 | .06 | 1.5 (0.98-2.29) | .10 | 1.44 (0.94-2.21) | .08 | 1.47 (0.95-2.26) |
|  | 65-75 | <.001 | 2.68 (1.52-4.74) | .002 | 2.45 (1.37-4.35) | .002 | 2.54 (1.42-4.54) |
| **Gender** | |  |  |  |  |  |  |
|  | Male | — | 1 | — | 1 | — | 1 |
|  | Female | <.001 | 1.59 (1.16-2.18) | .005 | 1.58 (1.15-2.17) | .007 | 1.55 (1.13-2.14) |
| **Education attainment** | |  |  |  |  |  |  |
|  | Basic | — | 1 | — | 1 | — | 1 |
|  | Medium | .88 | 1.04 (0.63-1.71) | .96 | 1.01 (0.61-1.67) | .87 | 0.96 (0.58-1.59) |
|  | Higher | .10 | 1.57 (0.91-2.70) | .15 | 1.49 (0.86-2.57) | .27 | 1.37 (0.79-2.37) |
| **Immigration history** | |  |  |  |  |  |  |
|  | Immigrants and their (direct) descendants | — | 1 | — | 1 | — | 1 |
|  | Individuals without immigration history | .23 | 1.4 (0.81-2.42) | .15 | 1.51 (0.87-2.63) | .10 | 1.59 (0.91-2.78) |
| **Residence** | |  |  |  |  |  |  |
|  | Rural area | — | 1 | — | 1 | — | 1 |
|  | Small town | .63 | 1.13 (0.69-1.83) | .75 | 1.08 (0.66-1.77) | .68 | 1.11 (0.68-1.82) |
|  | Medium-sized city | .93 | 1.02 (0.65-1.60) | .97 | 0.99 (0.63-1.56) | .96 | 1.01 (0.64-1.6) |
|  | Big city | .50 | 1.16 (0.76-1.77) | .72 | 1.08 (0.7-1.66) | .69 | 1.09 (0.71-1.68) |
| Vaccination readiness (scale) | |  |  | .007 | 1.17 (1.05-1.32) | .23 | 1.09 (0.95-1.25) |
| Knowledge score | |  |  |  |  | .055 | 1.19 (0.997-1,41) |
| Health literacy score | |  |  |  |  | .11 | 1.03 (0.99-1.07) |

Logistic regression on the characteristics of the *Infrequent and Passive Recipients*

| Variable | | Model 1 | | Model 2 | | Model 3 | |
| --- | --- | --- | --- | --- | --- | --- | --- |
|  | | *P value* | OR (95% CI) | *P value* | OR (95% CI) | *P value* | OR (95% CI) |
| **Age group (years)** | |  |  |  |  |  |  |
|  | 18-29 | — | 1 | — | 1 | — | 1 |
|  | 30-44 | 0.47 | 0.84 (0.52-1.35) | 0.45 | 0.83 (0.52-1.34) | 0.45 | 0.83 (0.51-1.35) |
|  | 45-64 | 0.19 | 0.74 (0.47-1.17) | 0.25 | 0.76 (0.48-1.21) | 0.26 | 0.76 (0.48-1.22) |
|  | 65-75 | 0.004 | 0.38 (0.2-0.73) | 0.01 | 0.41 (0.22-0.79) | 0.01 | 0.41 (0.21-0.78) |
| **Gender** | |  |  |  |  |  |  |
|  | Male | — | 1 | — | 1 | — | 1 |
|  | Female | 0.04 | 0.7 (0.5-0.99) | 0.05 | 0.7 (0.5-0.99) | 0.06 | 0.71 (0.5-1.01) |
| **Education attainment** | |  |  |  |  |  |  |
|  | Basic | — | 1 | — | 1 | — | 1 |
|  | Medium | 0.64 | 1.14 (0.65-1.99) | 0.59 | 1.17 (0.67-2.04) | 0.41 | 1.27 (0.72-2.23) |
|  | Higher | 0.54 | 0.83 (0.45-1.51) | 0.65 | 0.87 (0.47-1.59) | 0.88 | 0.96 (0.52-1.77) |
| **Immigration history** | |  |  |  |  |  |  |
|  | Immigrants and their (direct) descendants | — | 1 | — | 1 | — | 1 |
|  | Individuals without immigration history | 0.83 | 0.94 (0.52-1.69) | 0.68 | 0.88 (0.49-1.6) | 0.45 | 0.79 (0.43-1.45) |
| **Residence** | |  |  |  |  |  |  |
|  | Rural Area | — | 1 | — | 1 | — | 1 |
|  | Small town | 0.07 | 0.61 (0.36-1.04) | 0.09 | 0.63 (0.37-1.08) | 0.09 | 0.63 (0.37-1.08) |
|  | Medium-sized city | 0.49 | 0.84 (0.52-1.36) | 0.55 | 0.86 (0.54-1.39) | 0.57 | 0.87 (0.54-1.41) |
|  | Big city | 0.06 | 0.65 (0.41-1.02) | 0.1 | 0.68 (0.43-1.08) | 0.1 | 0.68 (0.43-1.08) |
| Vaccination readiness (scale) | |  |  | 0.04 | 0.88 (0.78-0.99) | 0.37 | 0.93 (0.81-1.08) |
| Knowledge score | |  |  |  |  | 0.21 | 0.89 (0.73-1.07) |
| Health literacy score | |  |  |  |  | <0.001 | 0.93 (0.89-0.96) |

Logistic regression on the characteristics of the *Frequent and Multi-Channel, Interaction-Focused Users*

| Variable | | Model 1 | | Model 2 | | Model 3 | |
| --- | --- | --- | --- | --- | --- | --- | --- |
|  | | *P value* | OR (95% CI) | *P value* | OR (95% CI) | *P value* | OR (95% CI) |
| **Age group (years)** | |  |  |  |  |  |  |
|  | 18-29 | — | 1 | — | 1 | — | 1 |
|  | 30-44 | 0.39 | 1.32 (0.7-2.46) | 0.4 | 1.31 (0.7-2.45) | 0.49 | 1.25 (0.66-2.36) |
|  | 45-64 | 0.35 | 0.74 (0.39-1.39) | 0.39 | 0.76 (0.4-1.43) | 0.31 | 0.72 (0.38-1.36) |
|  | 65-75 | 0.29 | 0.64 (0.28-1.46) | 0.36 | 0.68 (0.29-1.56) | 0.32 | 0.66 (0.28-1.51) |
| **Gender** | |  |  |  |  |  |  |
|  | Male | — | 1 | — | 1 | — | 1 |
|  | Female | 0.15 | 0.71 (0.45-1.12) | 0.16 | 0.72 (0.46-1.14) | 0.2 | 0.74 (0.47-1.17) |
| **Education attainment** | |  |  |  |  |  |  |
|  | Basic | — | 1 | — | 1 | — | 1 |
|  | Medium | 0.42 | 0.76 (0.39-1.49) | 0.44 | 0.77 (0.39-1.51) | 0.4 | 0.74 (0.38-1.47) |
|  | Higher | 0.12 | 0.55 (0.26-1.16) | 0.14 | 0.57 (0.27-1.2) | 0.14 | 0.57 (0.26-1.21) |
| **Immigration history** | |  |  |  |  |  |  |
|  | Immigrants and their (direct) descendants | — | 1 | — | 1 | — | 1 |
|  | Individuals without immigration history | 0.16 | 0.51 (0.2-1.3) | 0.14 | 0.49 (0.19-1.25) | 0.16 | 0.51 (0.19-1.32) |
| **Residence** | |  |  |  |  |  |  |
|  | Rural Area | — | 1 | — | 1 | — | 1 |
|  | Small town | 0.08 | 1.89 (0.92-3.91) | 0.07 | 1.95 (0.94-4.04) | 0.09 | 1.9 (0.91-3.97) |
|  | Medium-sized city | 0.4 | 1.36 (0.67-2.77) | 0.36 | 1.39 (0.68-2.84) | 0.44 | 1.33 (0.65-2.75) |
|  | Big city | 0.12 | 1.68 (0.87-3.24) | 0.1 | 1.76 (0.9-3.42) | 0.1 | 1.77 (0.9-3.48) |
| Vaccination readiness (scale) | |  |  | 0.24 | 0.91 (0.77-1.07) | 0.57 | 0.95 (0.78-1.14) |
| Knowledge score | |  |  |  |  | 0.27 | 0.88 (0.69-1.11) |
| Health literacy score | |  |  |  |  | 0.01 | 1.08 (1.02-1.13) |
